# Ruptured Abdominal Aortic Aneurysm Identified on Point-of-Care Ultrasound

**DOI:** 10.5811/cpcem.61897

**Published:** 2026-06-29

**Authors:** Michael H. Sherman

**Affiliations:** Boston University Chobanian and Avedisian School of Medicine, Boston Medical Center, Department of Emergency Medicine, Boston, Massachusetts

**Keywords:** abdominal aortic aneurysm, ruptured abdominal aortic aneurysm, point-of-care ultrasound, emergency medicine

## Abstract

**Case Presentation:**

A 78-year-old woman who presented to the emergency department with abdominal pain was found to have a tender, pulsatile abdominal mass on examination. Point-of-care ultrasound demonstrated an abdominal aortic aneurysm with findings concerning for active rupture. Emergent computed tomography angiography confirmed rupture with a large retroperitoneal hematoma.

**Discussion:**

Ruptured abdominal aortic aneurysm is a catastrophic diagnosis associated with high mortality, particularly when recognition is delayed. Point-of-care ultrasound is well established for identifying aneurysmal dilation; however, direct visualization of active rupture is rarely described.

## CASE PRESENTATION

A 78-year-old woman with a history of hypertension presented to the emergency department (ED) with acute lower abdominal pain. Initial vital signs demonstrated hemodynamic stability: blood pressure, 126/77 millimeters of mercury; heart rate, 87 beats per minute; respiratory rate, 18 breaths per minute; temperature, 36.9 ° C; and oxygen saturation, 100% on room air. On examination, the patient had a tender, pulsatile mass in the lower abdomen. Point-of-care ultrasound (POCUS) revealed a large, infrarenal abdominal aortic aneurysm with signs of active extraluminal leakage (Video), raising concern for rupture. Based on the physical examination and POCUS findings, vascular surgery was consulted emergently, and computed tomography angiography confirmed a ruptured abdominal aortic aneurysm with a large retroperitoneal hematoma (Image). The patient was emergently taken to the operating room for definitive surgical repair and was discharged after an uneventful recovery.

## DISCUSSION

Ruptured abdominal aortic aneurysm is a time-critical diagnosis with high mortality when recognition is delayed.^1^–^3^ While POCUS is routinely used in the ED to identify aneurysmal dilation, direct visualization of rupture is uncommon.^1^–^3^ In this case, POCUS (Panel A) demonstrated a markedly dilated infrarenal abdominal aorta with focal wall discontinuity and surrounding anechoic and heterogeneous fluid, findings consistent with active extravasation and retroperitoneal hemorrhage. Computed tomography angiography (Panel B) confirmed these findings, showing focal aortic wall disruption with an adjacent retroperitoneal hematoma.

This case highlights the rare demonstration of active abdominal aortic aneurysm rupture on POCUS and underscores the life-saving role of POCUS in rapidly identifying catastrophic vascular pathology.^2^–^4^ Recognition of wall disruption and retroperitoneal fluid on POCUS should prompt immediate surgical consultation, even in the absence of free intraperitoneal fluid. Early use of POCUS can expedite definitive imaging and operative intervention, significantly reducing time to life-saving care.

*CPC-EM Capsule*

What do we already know about this clinical entity?

*Ruptured abdominal aortic aneurysm (AAA) carries high mortality; Point-of-care ultrasound (POCUS) rapidly identifies aneurysmal dilation in unstable emergency department patients.*

What is the major impact of the image(s)?

*POCUS demonstrated focal aortic wall disruption with adjacent retroperitoneal fluid, suggesting active AAA rupture before computed tomography confirmation.*

How might this improve emergency medicine practice?

*Recognition of wall disruption and retroperitoneal fluid on POCUS should prompt immediate vascular surgery consultation and definitive care.*

## Supplementary Information

Video Real-time point-of-care ultrasound. Dynamic ultrasound clip of the abdominal aorta demonstrating a large aneurysm with a visible defect in the aortic wall (arrowhead).

## Figures and Tables

**Image:**
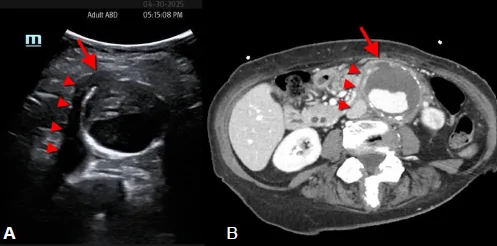
Point-of-care ultrasound and computed tomography angiography (A) Transabdominal longitudinal ultrasound view of the abdominal aorta showing a markedly dilated aneurysm with focal wall discontinuity (arrow) and adjacent hypoechoic collection (arrowheads). (B) Computed tomography angiography showing focal aortic wall disruption (arrow) with an adjacent retroperitoneal hematoma (arrowheads).
